# Investigation of the Mesiodistal Angulations of Maxillary Canines and Central Incisors for Missing Bilateral Maxillary Lateral Incisor

**DOI:** 10.3390/jcm13072110

**Published:** 2024-04-04

**Authors:** Orhan Cicek, Deniz Arslan

**Affiliations:** Department of Orthodontics, Faculty of Dentistry, Zonguldak Bulent Ecevit University, Zonguldak 67600, Türkiye; d.arslan@beun.edu.tr

**Keywords:** mesiodistal axial angulation, maxillary canine, maxillary central incisor, missing bilateral maxillary lateral incisor, space opening, space closure, orthodontic

## Abstract

**(1) Background:** A missing bilateral maxillary lateral incisor (MBMLI) causes aesthetic and functional problems and a multidisciplinary approach is required for treatment. This study aimed to compare the changes in the mesiodistal axial angulations of the maxillary canines and central incisors with orthodontic treatment of MBMLI. **(2) Methods:** A total of 56 patients with MBMLI were included in the study, and three groups were formed: the control group (Group 1, *n* = 20) with untreated ideal occlusion and the space opening (Group 2, *n* = 20) and space closure (Group 3, *n* = 16) groups as treated study groups. The mesiodistal angulations between the long axes of the maxillary right canine (tooth no 13), right central incisor (tooth no 11), left central incisor (tooth no 21) and maxillary left canine (tooth no 23), and the bicondylar plane, were measured on the panoramic radiographs taken pre (T0) and post treatment (T1). *p* < 0.05 was accepted for statistical significance. **(3) Results:** At T0, while there was no significant difference between the mesiodistal angulations of the right–left maxillary canines and central incisors in all groups (*p* > 0.05), the mesiodistal angulations of the canines in the Group 1 were significantly higher than the study groups (*p* < 0.05). With treatment, while the mesiodistal angulation of the canines increased in Group 2, it decreased in Group 3 (*p* < 0.05). On the other hand, the mesiodistal angulation of the central incisors decreased in Group 2 and did not change in Group 3 (*p* > 0.05). At T1, the mesiodistal angulation of the canines was found to be lower in Group 3 than in Groups 1 and 2, while the angulation of the central incisors was found to be lower in Group 2 compared to Group 1 (*p* < 0.05). **(4) Conclusions:** In the orthodontic treatment of MBMLIs, changes in the mesiodistal angulations of the maxillary canine and central incisors should be taken into account for satisfactory outcomes. It was concluded that there should be a tendency to select the space closure method in which normal mesiodistal angulations are obtained in maxillary central incisors for aesthetics and planned incisor position, and also at a low cost.

## 1. Introduction

The mesiodistal axial angulations of the maxillary anterior teeth play a critical role in ensuring ideal occlusion and the maintenance of normal function. Because the mesiodistal angulation of the incisors influences their placement on the dental arch, the mesiodistal angulation of the anterior teeth controls the location of the posterior teeth in the arch, and hence the posterior occlusion [1]. In addition, since the maxillary anterior teeth are the main determinant of the smile, these teeth have a high potential to increase the functional aesthetics and attractiveness of the individual through orthodontic tooth movement [2,3].

In order to achieve the occlusal standards targeted by orthodontic treatment, the teeth should be positioned correctly in three dimensions of the space [4]. For this reason, the ideal alignment of the teeth by positioning appropriate mesiodistal axial angulations is also important for the stability of orthodontic treatment results [5]. Also, the American Board of Orthodontics has reported that panoramic radiography can be used to evaluate root parallelism and mesiodistal axial angulations during the orthodontic treatment process [6].

A missing bilateral maxillary lateral incisor MBMLI, which is not accompanied by the absence of any other teeth other than the third molars, is called mild hypodontia [7]. Patients with MBMLI seek orthodontic treatment with high aesthetic expectations due to the anterior location of the malocclusion [8]. It is still controversial in the literature whether MBMLIs should be treated with space opening or closure [8,9], and in its treatment: (1) if primary lateral incisors are present, they should be kept together with diastemas; (2) space closure, which includes moving the canines to the lateral incisor position and first premolars to the canine tooth place; or (3) space opening methods for prosthetic rehabilitation, are applied [7]. While it is obvious what the first method will do, the second and third methods may also cause compromises in aesthetics, periodontal health and function [10].

Missing bilateral maxillary lateral incisors, which are reported to be among the most common developmental dental anomalies, are reported to be more common in Asians and females, and their prevalence is between 1.5 and 35.6% [11,12,13]. It has been associated with a variety of factors, including ectodermal dysplasia, Down syndrome, cleft lip and palate, medication side effects, trauma, root resorption, infection, and gene abnormalities (MSX and PAX9) [14,15]. An interdisciplinary approach focusing on smile, function, and aesthetics rehabilitation is necessary for its treatment, which calls for collaboration between the orthodontists, prosthodontists, restorative dentists and oral surgeons [7,12,16].

After tooth loss or in cases of congenital tooth absence, buccolingual width and vertical height losses occur in the alveolar bone [17]. It has been reported that there is a decrease in the buccolingual width of the alveolar bone after the treatment of MBMLI with space opening [18,19]. Although it has been reported that this decrease is stable during the retention period [20], these reductions, especially in the buccal alveolar region, need to be supported with bone grafting or dental implants should be placed more palatally [17,21]. On the other hand, in space closure, the concave profile would become evident as the midface deficiency increased and the enamel of the canine would have to be grinded and reshaped with composite to make it resemble a normal lateral incisor [8]. Thus, considering the possible disadvantages expected with both methods, an individual treatment plan needs to be constituted after a careful diagnosis and a comprehensive interdisciplinary consultation [22].

Although there are studies investigating the relationship between the mesiodistal angulations of the maxillary lateral incisors and gender, different age groups, functional occlusal plane, and aesthetics [23,24], no study has been found investigating the mesiodistal angulations of the maxillary canine and central incisors in the MBMLIs. Moreover, when the literature is examined, although there are studies on the relationship of the mesiodistal angulations of various teeth with different facial growth patterns, different types of orthodontic malocclusion or third molars [25,26,27], the presented study represents research on the subject from a different aspect. In this context, the presented study is the first research that seeks answers to which method could be used for aesthetic and stable occlusal goals in the orthodontic treatment of MBMLI, based on the mesiodistal axial angulation changes that occur in the maxillary canine and central incisors.

Therefore, the presented study aimed to compare the changes in the mesiodistal axial angulations of the maxillary canine and central incisors in congenital MBMLIs treated orthodontically with space opening and closure methods, with the control group having ideal occlusal relationships, using panoramic radiographs. The null hypothesis of the study is that there is no difference between the groups in the mesiodistal axial angulations of the maxillary canine and central incisors.

## 2. Materials and Methods

The subjects of the study consisted of patients with MBMLI who applied to Zonguldak Bülent Ecevit University Orthodontics Department for orthodontic treatment. Prior to the study, ethical approval was obtained from Zonguldak Bülent Ecevit University Non-Interventional Clinical Research Ethics Committee (No: 2024/03-1).

In the study, the sample size calculation was performed with G*Power program (version 3.1.9.7; Franz Faul, Universität Kiel, Kiel, Germany). The ‘tests—Means: Difference between two dependent means (matched pairs)’ was selected and the one-way hypothesis (Tail(s): one) was determined. When the power of the study (1—β error probability) was determined as 0.95 and the α error prob was determined as 0.05, the effect size was calculated as 0.44. Accordingly, when at least a total of 46 samples were included, the actual power of the study was calculated as 90%. In the study, a total of 56 patients were included, and 3 groups were formed: a control group (*n* = 20, Group 1) that did not receive orthodontic treatment, and space opening (*n* = 20, Group 2) and space closure (*n* = 16, Group 3) study groups. Data on patients’ age, gender and total treatment duration are shown in Table 1.

In the first step of the study, the files of patients with MBMLI who had completed fixed orthodontic treatment were scanned and examined from the clinic archives. As a result of the archive scanning, the follow-up files of the patients whose orthodontic treatment was completed with space opening and closure methods were evaluated separately. Patients were divided according to the inclusion criteria, taking into account the space opening and closure methods. Inclusion criteria for the study groups (Group 2 and Group 3) were as follows:

- Having congenital bilateral maxillary lateral incisor absence;
- Not having skeletal problems;
- Not having prior orthodontic treatment;
- Fixed orthodontic treatment completed with 0.22 inch slot MBT^TM^ prescription;
- In the space opening group, having canines with the root tips positioned closer to the original position, more distal from the midline and having no or mild crowding and/or diastemas (<2 mm) in the mandible;
- In the space closure group, having canines with the root tips positioned more mesially than its original position, closer to the midline and having no or mild crowding and/or diastemas (<2 mm) in the mandible;
- Having radiographs with high resolution and good image quality.

Inclusion criteria for the untreated control group (Group 1) patients with ideal occlusal characteristics were as follows:

- No need for orthodontic treatment;
- Not having skeletal problems
- Having class I molar and canine relationship;
- No missing teeth except third molars;
- Having no or mild crowding and/or diastemas (<2 mm);
- Having normal overjet and overbite;
- Having radiographs with high resolution and good image quality.

Patients who did not meet at least one of the inclusion criteria were excluded from the study. The lateral cephalograms of the patients were taken with a cephalometric X-ray machine (Veraviewepocs 2D, J Morita Mfg. Corp., Kyoto, Japan). SNA, SNB, ANB and SN/GoGn skeletal angular measurements on lateral cephalometric radiographs were made in the NemoCeph (Nemotec, 2020, Madrid, Spain) cephalometric analysis program. In Table 2, data on these normal skeletal angular parameters for the included patients are given separately in each group.

**Figure 1:**
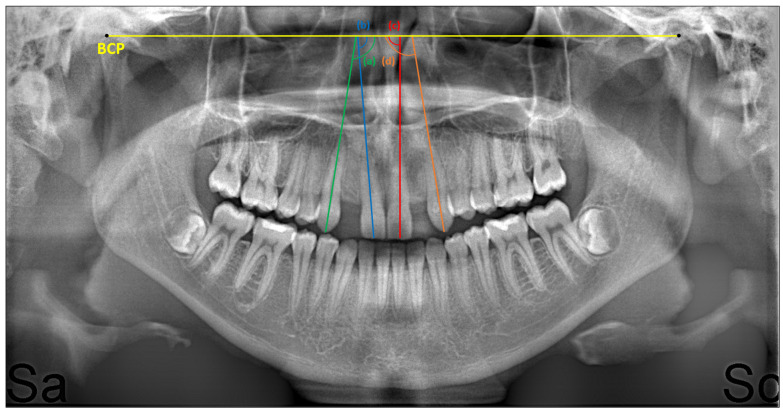
Mesiodistal axial angulations of a patient measured after space opening: (a) for tooth no 13, (b) for tooth no 11, (c) for tooth no 21, and (d) for tooth no 23. BCP: Bicondylar plane.

The mesiodistal axial angulations of the right maxillary canine (tooth no 13), right maxillary central incisor (tooth no 11), left maxillary central incisor (tooth no 21) and left maxillary canine teeth (tooth no 23) were measured on the panoramic radiographs taken pre- (T0) and post-treatment (T1). While taking panoramic radiographs on the X-ray machine (Veraviewepocs 2D, J Morita Mfg. Corp., Kyoto, Japan), care was taken to ensure that the Frankfurt horizontal plane was parallel to the ground and that the bite stick was bitten in the correct position. The panoramic radiographs were transferred to the NemoCeph analysis program (Nemotec, 2020, Madrid, Spain) for measurements. During the measurement, the bicondylar plane drawn from the top and front points of the right and left condyles on the panoramic radiograph was created as a fixed reference [28]. The medial angle between this reference line and the long axis passing through the cusp tip for maxillary canines and the midpoint of the incisal edge for maxillary central incisors was measured, and recorded. Measurements were performed manually and recorded separately at T0 and T1 in the study groups, and in the control group (see Figure 1).

### 2.1. Orthodontic Treatment Protocols for Group 2 and Group 3 Study Groups

**Figure 2:**
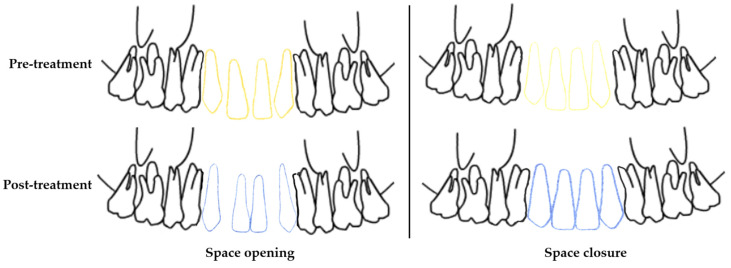
Schematic diagrams of the movements of the maxillary canines and central incisors in the pre- and post-treatment (Yellow lines: positions of the maxillary canines and central incisors in the pre-treatment. Blue lines: positions of the maxillary canines and central incisors in the post-treatment).

Fixed orthodontic treatments were carried out using stainless steel metal brackets with a 0.022 × 0.028 inch slot MBT^TM^ prescription (Mini Master, American Orthodontics, Sheboygan, WI, USA) [29]. During the sessions, round 0.014 inch heat-activated nickel-titanium (HANT), 0.016 inch HANT, and 0.016 inch stainless steel arch wires were applied to the patients, followed by rectangular 0.019 × 0.025 inch HANT, and rectangular 0.019 × 0.025 stainless steel arch wires (American Orthodontics, USA). In the space opening group, after 1 session of applying rectangular 0.019 × 0.025 stainless steel arch wire, open coil springs were placed and activated and space opening was applied. Orthodontic treatment was completed after the space obtained for prosthetic rehabilitation of the maxillary lateral incisor was approved by prosthodontist and surgeon consultation. In the space closure group, a 0.019 × 0.025 stainless rectangular arch wire with hook (Brass posted, American Orthodontics, USA) was placed following the 0.019 × 0.025 HANT (American Orthodontics) arch wire and we waited for 1 session. Then, a 1.6 × 8 mm miniscrew temporary anchorage device (Aarhus System, American Orthodontics) was placed in the interdental alveolar area at the mid-root level of the maxillary central incisors under local anesthesia. The central incisors were tied to this miniscrew with a steel wire ligature. Fixed orthodontic treatment was completed by applying tieback mechanics with indirect anchorage and closing the space with minimum anchorage. The schematic diagram of orthodontic movements in the teeth is shown in Figure 2.

### 2.2. Statistical Analysis

SPSS version 26 program (IBM Corporation, New York, NY, USA) was used for statistical analysis of the data. Normality distribution was evaluated with the Shapiro–Wilk test. Accordingly, since the data showed normal distribution, a paired sample t-test was used for intra-group comparisons, and one-way ANOVA and post hoc Tukey tests were used for inter-group comparisons. The reliability test between the measurements performed after 4 weeks on 10 randomly selected panoramic radiographs was evaluated with the intraclass correlation coefficient. *p* < 0.05 was accepted for statistical significance.

## 3. Results

Intraclass correlation coefficients were found to be between 0.90 and 0.97 in repeated measurements, revealing high intra-observer reliability.

In Group 1, no statistically significant differences were found between the mesiodistal angulation of the maxillary right-left canine and central incisors (*p* > 0.05). Similarly, no statistically significant difference was found between the mesiodistal angulations of the maxillary right-left canine and central incisors in Groups 2 and 3, both at T0 and T1 (*p* > 0.05). The statistical analysis results of the right-left canine and central incisor mesiodistal angulations in each group are given in Table 3.

In Group 2, after treatment, the mesiodistal angulations of teeth 13 and 23 increased significantly, while those of teeth 11 and 21 decreased (*p* < 0.05). In Group 3, while the mesiodistal angulations of teeth 13 and 23 decreased significantly after treatment, it was observed that there was no significant change in teeth 11 and 21. Paired-samples *t* test results for the data of Group 2 and Group 3, where space opening and closure were applied, respectively, are shown in Table 4.

In period T0:

The mesiodistal angulations of tooth 13 was found to be statistically significantly higher in Group 1 than in Group 3 (*p* < 0.05). There was no significant difference between Group 1 and Group 2 and between Group 2 and Group 3 (*p* > 0.05). The mesiodistal angulations of tooth 23 was found to be significantly higher in Group 1 than in Group 2 and Group 3 (*p* < 0.05). However, no significant difference was found between Group 2 and Group 3 (*p* > 0.05). There was no significant difference between the groups in the mesiodistal angulations of teeth 11 and 21 (*p* > 0.05).

In period T1:

While the mesiodistal angulations of teeth 13 and 23 were found to be significantly lower in Group 3 than in Group 1 and Group 2 (*p* < 0.05), there was no significant difference between Group 1 and Group 2 (*p* > 0.05). The mesiodistal angulations of teeth 11 and 21 were found to be significantly lower in Group 2, where space opening treatment was applied, than in Group 1 (*p* < 0.05). However, the mesiodistal angulations of teeth 11 and 21 did not show a significant difference both between Group 1 and Group 3 and between Group 2 and Group 3 (*p* > 0.05).

One-way ANOVA results for inter-group comparisons at T0 and T1 for each tooth are shown in Table 5.

## 4. Discussion

Mesiodistal axial angulations and inclinations have been a long-researched topic in orthodontics, and considering the geometric shape of the anterior dental arch, it has been reported that mesiodistal angulations of incisors and canines should continue to be investigated [30,31]. The presented study revealed that the differences in the mesiodistal axial angulations of the maxillary canine and central incisors caused by two different orthodontic treatment approaches applied to MBMLI and that the planned final incisor positions should be carefully evaluated during pre-treatment planning.

In the literature, it has been reported that panoramic radiographs can be used in the evaluation of root inclination and parallelism and in the evaluation of axial angulations with a reference plane, and that they have advantages such as causing less radiation and being inexpensive [27,32,33]. Therefore, in this study, the mesiodistal angulations of the maxillary canine and central incisors were measured according to the bicondylar reference line on panoramic radiographs.

In the rehabilitation of patients with MBMLI, there are options to close the orthodontic space by reshaping the canines by repositioning them mesially or to open the space and intervene with an implant/tooth-supported prosthesis. However, there has been no study comparing which of the two methods is superior in terms of biological, functional and aesthetics [34]. In this sense, in our study, remarkable results were observed in the mesiodistal angulation of the maxillary canines and central incisors for the planned incisor position and root parallelism, which are necessary to achieve aesthetically and functionally stable orthodontic results in MBMLI. In the treatment of patients with MBMLI with the space opening method, we observed that the mesiodistal axial angulations of the canines increased, while space closure decreased. The mesiodistal angulations of the central incisors showed a significant decrease in the space opening group. On the other hand, although an increase in the angulation of the central incisors was observed in the space closure group, this increase was not found to be significant.

Barakaat et al. [35], in their study investigating the differences in mesiodistal root angulation, reported that they did not find a significant difference between panoramic–cone beam computed tomography (pan-CBCT) and CBCT images in terms of mesiodistal root angulation, and in terms of sexual dimorphism, females showed more mesial angulation than males. In our study, more mesial tipping and mesiodistal angulation decreasing were observed in the central incisors in space opening and in the canines in space closure. Additionally, previous studies have reported that the frequency of congenital MBMLI is 1.5 to 2.0 times higher in females than in males [34,36,37]. There was no gender group in our study, but it was compatible with the literature since the majority of the MBMLI sample consisted of females.

It is known that the mesiodistal angulation of the maxillary anterior teeth is not only related to occlusal goals, but also to aesthetic appeal [1,3]. It has been reported in a previous study that moving the maxillary anterior teeth has the potential to increase smile attractiveness [2], and it was reported by Yang et al. [3] that a positive increase in the mesiodistal angulation of the maxillary central incisors in the mesial direction causes a more attractive smile than a negative increase in the distal direction. In our study, in the space opening Group 2, the maxillary central incisors angulations decreased and a tendency towards mesial tipping was observed. It is thought that the reason why the decrease in mesiodistal axial angulation due to the tipping of the central incisors to the mesial is found to increase facial attractiveness is due to the fact that the canines are moved towards their original place during the space opening and thus all teeth are positioned in the ideal position. However, it should be kept in mind that only these angulation improvements in the central incisor are not sufficient for aesthetics, and the implantological/prosthetic rehabilitation planned for the lateral incisor should also be compatible with this. In addition, even if the orthodontic space opening treatment is completed successfully in all aspects, undesirable situations ranging from minor problems to re-orthodontic treatment may be encountered, especially during the period when individuals in adolescence have to wait until adulthood for implant insertion [38]. In our study, the mean age of the patients to whom we applied space opening was 18 and they were suitable for implant insertion without wasting time after orthodontic treatment.

There are also other studies in the literature that have investigated the orthodontic treatment of MBMLI using space opening and closure methods, both aesthetically and functionally [16,18,39]. Josefsson et al. [12], in their study investigating whether implant treatment or orthodontic space closure is the best long-term treatment option for patients with MBMLIs, reported that there were some disadvantages regarding clinical crown and aesthetics in both space opening and closure groups. They reported that if both methods can be applied, the space closure method should be preferred and some factors such as the inclination of the incisors, profile, occlusion, whether there is available space in the dental arch and gingival appearance should be taken into consideration during treatment planning. Jamilian et al. compared the aesthetic, periodontal, and functional outcomes of patients with MBMLI five years after treatment with space closure and implant substitution. In their study, the authors reported similar satisfactory aesthetic results with both treatment methods, but also reported that a significant infraocclusion was observed with the implants and periodontal health was better with the space closure [38]. In another study, it was reported that there was no significant difference between the space opening and closing groups in terms of plaque accumulation and bleeding during probing [40].

In the presented study, orthodontic treatment was completed successfully with both methods, and considerable changes occurred in the mesiodistal axial angulations of the maxillary canine and central incisors after the treatment. The decrease in mesiodistal angulation due to mesial tipping in the central incisors may raise doubts about the root parallelism, which is crucial for the planned incisor position, even though canine angulations in the space opening group approached the control group and space was successfully created for the implant insertion. On the other hand, in the space closure group, central incisor angulation approached normal and there was no significant difference between it and the control group, which was found to be clinically important. These results of our study revealed that, based on these angulation changes, the space closure method may be more suitable for patients with MBMLI.

In the literature, studies on the method by which MBMLI is mostly treated have reported that the space closure method is 87.5% more common than space opening [34,41]. However, clear indications for the space opening method, in which spaces mesial and distal to the canines and central incisors are managed for occlusion and aesthetic goals, have been reported as Class I molar cases with no malocclusion, Class III malocclusions with a concave facial profile, and cases in which reshaping of the canines is not recommended [34,42]. In the presented study, attention was paid to the homogeneous distribution of the groups to ensure reliable findings that answered the aim of the research. For this purpose, patients with skeletally normal angles were included due to the potential for skeletal differences to affect mesiodistal angulation changes in the teeth. However, the findings of our study revealed that, if possible, there should be a tendency towards the space closure method in terms of aesthetics, functionality and cost, because the root parallelism required for the planned central incisor position and the desired mesiodistal angulation changes for aesthetic goals were achieved in the space closure method.

The limitation of the study is that since there was no equivalent study with a similar sample group, not many associations or comparisons with other studies could be made. Other limitations include not examining differences in mesiodistal axial angulations according to gender and soft tissues. For this, further studies need to be planned in larger standardized sample groups. However, this presented study provides important and different results regarding the changes in the mesiodistal axial angulation of the maxillary canines and central incisors with the treatment of MBMLI.

## 5. Conclusions

- The null hypothesis of the study was rejected because differences were found between the groups in the mesiodistal axial angulations of the maxillary canines and central incisors in pre- and post-treatment;
- Since normal mesiodistal axial angulations are targeted in orthodontic treatment and the treatment of MBMLI is possible with both methods, it is recommended that orthodontists include these mesiodistal axial angulation changes in planning when determining the planned incisor tooth positions in treatment planning;
- It was concluded that there should be a tendency towards the space closure method, which eliminates the anatomical disadvantages of the alveolar bone in cases where a space is opened, ensures the planned incisor position, and offers a low cost advantage by eliminating the waiting for the subsequent implant insertion.

## Figures and Tables

**Table 1:** Data on patients’ age, gender and total treatment duration.

		Group 1	Group 2	Group 3
Age	Mean ± SD	17.3 ± 3.9	18.0 ± 4.5	15.6 ± 3.5
Gender	Female (*n*—%)	16–80%	16–80%	13–81.2%
	Male (*n*—%)	4–20%	4–20%	3–18.8%
Treatment duration (year, Mean ± SD)			2.2 ± 0.6	3.1 ± 1.02

SD: standard deviation, *n*: sample size, %: percentage

**Table 2:** Data on patients’ skeletal angular measurements.

		Group 1	Group 2	Group 3
SNA	Mean ± SD	81.56 ± 1.5	80.9 ± 2.9	79.2 ± 2.8
SNB	Mean ± SD	79.4 ± 1.1	79.3 ± 2.5	77.4 ± 3.1
ANB	Mean ± SD	2.2 ± 1.1	1.7 ± 1.0	1.8 ± 1.3
SN/GoGn	Mean ± SD	33.9 ± 2.8	33.1 ± 3.4	34.7 ± 3.6

SD: standard deviation.

**Table 3:** Independent sample *t* test results for maxillary canine and central incisors angulations in all groups.

		Tooth No	*n*	Mean ± SD	*p*
Group 1 (Control)		13	20	92.69 ± 5.26	0.72
		23	20	93.18 ± 3.07	
		11	20	90.14 ± 2.69	0.33
		21	20	90.95 ± 2.53	
Group 2 (Space opening)	T0	13	20	89.04 ± 5.23	0.93
		23	20	88.89 ± 5.14	
	T1	13	20	93.71 ± 4.81	0.57
		23	20	92.86 ± 4.50	
	T0	11	20	88.92 ± 2.58	0.51
		21	20	89.68 ± 4.38	
	T1	11	20	87.27 ± 3.21	0.76
		21	20	86.92 ± 4.09	
Group 3 (Space closure)	T0	13	16	88.70 ± 4.03	0.82
		23	16	89.07 ± 5.23	
	T1	13	16	85.51 ± 3.95	0.81
		23	16	85.15 ± 4.47	
	T0	11	16	88.23 ± 2.29	0.66
		21	16	88.67 ± 3.31	
	T1	11	16	89.12 ± 3.62	0.98
		21	16	89.10 ± 2.85	

T0: pre-treatment, T1: post-treatment, *n*: sample size, SD: standard deviation, *p*: significance level.

**Table 4:** Paired-samples *t* test results for the changes during treatment in the study groups.

	Tooth No		T0	T1	*p*
Group 2 (Space opening)					
	13	Mean ± SD	89.04 ± 5.23	93.71 ± 4.81	0.004 *
	23	Mean ± SD	88.89 ± 5.14	92.86 ± 4.50	0.015 *
	11	Mean ± SD	88.92 ± 2.58	87.27 ± 3.21	0.02 *
	21	Mean ± SD	89.68 ± 4.38	86.92 ± 4.09	0.02 *
Group 3 (Space closure)					
	13	Mean ± SD	88.70 ± 4.03	85.51± 3.95	0.03 *
	23	Mean ± SD	89.07 ± 5.23	85.15 ± 4.47	0.01 *
	11	Mean ± SD	88.23 ± 2.29	89.12 ± 3.62	0.46
	21	Mean ± SD	88.67 ± 3.31	89.10 ± 2.85	0.52

T0: pre-treatment, T1: post-treatment, SD: standard deviation, *p*: significance level, *: *p* < 0.05.

**Table 5:** One-way ANOVA results of inter-group comparisons for each tooth in T0 and T1.

	Tooth No		Group 1 ^a^	Group 2 ^b^	Group 3 ^c^	*p*
T0						
	13	Mean ± SD	92.69 ± 5.26 ^c^	89.04 ± 5.28	88.70 ± 4.03	0.028 *
	23	Mean ± SD	93.18 ± 3.07 ^b,c^	88.89 ± 5.14	89.07 ± 5.23	0.007 *
	11	Mean ± SD	90.14 ± 2.69	88.92 ± 2.58	88.23 ± 2.29	0.08
	21	Mean ± SD	90.95 ± 2.53	89.68 ± 4.38	88.67 ± 3.31	0.15
T1						
	13	Mean ± SD	92.69 ± 5.26	93.71 ± 4.81	85.51 ± 3.95 ^a,b^	<0.001 *
	23	Mean ± SD	93.18 ± 3.07	92.86 ± 4.50	85.15 ± 4.47 ^a,b^	<0.001 *
	11	Mean ± SD	90.14 ± 2.69	87.27 ± 3.21 ^a^	89.12 ± 3.62	0.02 *
	21	Mean ± SD	90.95 ± 2.53	86.92 ± 4.09 ^a^	89.10 ± 2.85	0.001 *

T0: pre-treatment, T1: post-treatment, SD: standard deviation, *p*: significance level, *: *p* < 0.05. ^a^: Difference with Group 1, *p* < 0.05, ^b^: Difference with Group 2 *p* < 0.05, ^c^: Difference with Group 3 *p* < 0.05.

## Data Availability

All data supporting the results of this study are included within the article. The data are currently not publicly available as they will be used in another study still in progress.

## References

[1] De Almeida-Pedrin R.R., Pinzan A., de Almeida R.R., Ursi W., de Almeida M.R. (2006). Panoramic evaluation of mesiodistal axial inclinations of maxillary anterior teeth in orthodontically treated subjects. Am. J. Orthod. Dentofac. Orthop..

[2] Sarver D.M. (2001). The importance of incisor positioning in the esthetic smile: The smile arc. Am. J. Orthod. Dentofac. Orthop..

[3] Yang S., Guo Y., Yang X., Zhang F., Wang J., Qiu J., Li J. (2015). Effect of mesiodistal angulation of the maxillary central incisors on esthetic perceptions of the smile in the frontal view. Am. J. Orthod. Dentofac. Orthop..

[4] Andrews L.F. (2015). The 6-elements orthodontic philosophy: Treatment goals, classification, and rules for treating. Am. J. Orthod. Dentofac. Orthop..

[5] Fontana M., Fastuca R., Zecca P.A., Nucera R., Militi A., Lucchese A., Portelli M., Caprioglio A. (2021). Correlation between Mesio-Distal Angulation and Bucco.-Lingual Inclination of First and Second Maxillary Premolars Evaluated with Panoramic Radiography and Cone-Beam Computed Tomography. Appl. Sci..

[6] American Board of Orthodontics (2008). Grading System for Dental Casts and Panoramic Radiographs.

[7] Westgate E., Waring D., Malik O., Darcey J. (2019). Management of missing maxillary lateral incisors in general practice: Space opening versus space closure. Br. Dent. J..

[8] Oliveira D.D., de Oliveira B.F., da Mata Cid Pinto L.S., Figueiredo D.S.F., Pithon M.M., Seraidarian P.I. (2013). Interdisciplinary Treatment of a C lass III Patient with Congenitally Absent Maxillary Lateral Incisors. J. Esthet. Restor. Dent..

[9] Kinzer G.A., Kokich Jr V.O. (2005). Managing congenitally missing lateral incisors. Part II: Tooth-supported restorations. J. Esthet. Restor. Dent..

[10] Abu-Hussein M., Watted N., Abdulgani A., Borbély P. (2015). Modern treatment for congenitally missing teeth: A multidisciplinary approach. Int. J. Oral Maxillofac. Surg..

[11] Gupta S.P., Rauniyar S. (2021). Management of missing maxillary lateral incisor: A contemporary review. Orthod. J. Nepal.

[12] Josefsson E., Lindsten R. (2019). Treatment of missing maxillary lateral incisors: A clinical and aesthetic evaluation. Eur. J. Orthod..

[13] Vahid-Dastjerdi E., Borzabadi-Farahani A., Mahdian M., Amini N. (2010). Non-syndromic hypodontia in an Iranian orthodontic population. J. Oral Sci..

[14] Mostowska A., Kobielak A., Biedziak B., Trzeciak W.H. (2003). Novel mutation in the paired box sequence of PAX9 gene in a sporadic form of oligodontia. Eur. J. Oral Sci..

[15] Ericson S., Kurol J. (2000). Resorption of incisors after ectopic eruption of maxillary canines: A CT study. Angle Orthod..

[16] Turpin D.L. (2004). Treatment of missing lateral incisors. Am. J. Orthod. Dentofac. Orthop..

[17] Borzabadi-Farahani A. (2012). Orthodontic considerations in restorative management of hypodontia patients with endosseous implants. J. Oral Implantol..

[18] Uribe F., Chau V., Padala S., Neace W.P., Cutrera A., Nanda R. (2013). Alveolar ridge width and height changes after orthodontic space opening in patients congenitally missing maxillary lateral incisors. Eur. J. Orthod..

[19] Nováčková S., Marek I., Kamínek M. (2011). Orthodontic tooth movement: Bone formation and its stability over time. Am. J. Orthod. Dentofac. Orthop..

[20] Ostler M.S., Kokich V.G. (1994). Alveolar ridge changes in patients congenitally missing mandibular second premolars. J. Prosthet. Dent..

[21] Borzabadi-Farahani A., Zadeh H.H., Tolstunov L. (2016). Orthodontic therapy in implant dentistry: Orthodontic implant site development. Vertical Alveolar Ridge Augmentation in Implant Dentistry: A Surgical Manual.

[22] Araújo E.A., Oliveira D.D., Araújo M.T. (2006). Diagnostic protocol in cases of congenitally missing maxillary lateral incisors. World J. Orthod..

[23] Hajira N., Shashi Dhara H., Khandelwal P. (2022). Assessment of Mesiodistal Angulation of Maxillary Lateral Incisors: A Cross-sectional Study. J. Clin. Diagn. Res..

[24] Ueda H., Masunaga M., Horie K., Medina C., Tanne K. (2016). Mesiodistal angulation of the lateral teeth to the functional occlusal plane in normal occlusions. APOS Trends Orthod..

[25] Cuoghi O.A., Sella R.C., De Mendonça M.R. (2010). Mesiodistal angulations of the mandibular canines, premolars and molars with or without the presence of third molars. Eur. J. Orthod..

[26] Badiee M., Ebadifar A., Sajedi S. (2019). Mesiodistal angulation of posterior teeth in orthodontic patients with different facial growth patterns. J. Dent. Res. Dent. Clin. Dent. Prospect..

[27] Cicek O., Yilmaz H., Demir Cicek B. (2023). Comparison of the Mesiodistal Angulations of Canine and Molar Teeth in Different Types of Orthodontic Malocclusions: A Retrospective Study. Diagnostics.

[28] Cicek O., Gurel T., Demir Cicek B. (2023). Investigation of the Relationship of Impacted Maxillary Canines with Orthodontic Malocclusion: A Retrospective Study. Children.

[29] McLaughlin R.P., Bennett J.C., Trevisi H. (2001). Systemized Orthodontic Treatment Mechanics.

[30] Ohashi A.S.C., Nascimento K.C.G.d., Normando D. (2011). Analysis of the correlation between mesiodistal angulation of canines and labiolingual inclination of incisors. Dent. Press. J. Orthod..

[31] Busato M.C.A., Mendonça M.R.d., Pereira A.L.P., Tondelli P.M., Cuoghi O.A. (2009). Compensatory canine angulation in angle Class II and III patients. Braz. Oral Res..

[32] Garcia-Figueroa M.A., Raboud D.W., Lam E.W., Heo G., Major P.W. (2008). Effect of buccolingual root angulation on the mesiodistal angulation shown on panoramic radiographs. Am. J. Orthod. Dentofac. Orthop..

[33] Rocha C.A., Almeida R.R.d., Henriques J.F.C., Flores-Mir C., Almeida M.R.d. (2016). Evaluation of long-term stability of mesiodistal axial inclinations of maxillary molars through panoramic radiographs in subjects treated with Pendulum appliance. Dent. Press. J. Orthod..

[34] Kiliaridis S., Sidira M., Kirmanidou Y., Michalakis K. (2016). Treatment options for congenitally missing lateral incisors. Eur. J. Oral Implantol..

[35] Barakaat A.A., Maaz M., Sukhia R.H., Fida M. (2023). Comparison of mesiodistal root angulation of teeth by conventional panoramic and cone beam computed tomography images–A cross-sectional study. Int. Orthod..

[36] Polder B.J., Van’t Hof M.A., Van der Linden F.P., Kuijpers-Jagtman A.M. (2004). A meta-analysis of the prevalence of dental agenesis of permanent teeth. Community Dent. Oral Epidemiol..

[37] Pinho T., Tavares P., Maciel P., Pollmann C. (2005). Developmental absence of maxillary lateral incisors in the Portuguese population. Eur. J. Orthod..

[38] Jamilian A., Perillo L., Rosa M. (2015). Missing upper incisors: A retrospective study of orthodontic space closure versus implant. Prog. Orthod..

[39] Beyer A., Tausche E., Boening K., Harzer W. (2007). Orthodontic space opening in patients with congenitally missing lateral incisors: Timing of orthodontic treatment and implant insertion. Angle Orthod..

[40] De Marchi L.M., Pini N.I.P., Hayacibara R.M., Silva R.S., Pascotto R.C. (2012). Congenitally missing maxillary lateral incisors: Functional and periodontal aspects in patients treated with implants or space closure and tooth re-contouring. Open Dent. J..

[41] Fekonja A. (2005). Hypodontia in orthodontically treated children. Eur. J. Orthod..

[42] Rosa M., Zachrisson B.U. (2001). Integrating esthetic dentistry and space closure in patients with missing maxillary lateral incisors. J. Clin. Orthod..

